# Cardiac Cachexia Associated With Valvular Heart Failure

**DOI:** 10.7759/cureus.20109

**Published:** 2021-12-02

**Authors:** Miguel A Rodriguez-Guerra, Neelanjana Pandey, Timothy J Vittorio

**Affiliations:** 1 Medicine, Montefiore Medical Center, Albert Einstein College of Medicine, New York, USA; 2 Internal Medicine, BronxCare Health System, Icahn School of Medicine at Mount Sinai, New York, USA; 3 Cardiology, BronxCare Health System, Icahn School of Medicine at Mount Sinai, New York, USA

**Keywords:** tavr, aortic stenosis, valvulopathy, heart failure, cardiac cachexia

## Abstract

Cardiac cachexia (CC) represents a serious complication of heart failure (HF). This condition could be directly related to mortality. The weight or muscle mass loss has to be monitored in our patients with HF to avoid potential complications.

We report a case of an elderly patient with a history of aortic stenosis (AS) who presented with progressive shortness of breath limiting his daily activities associated with weight loss. Signs of heart failure were evident on physical examination, and valvulopathy was also evident. His echocardiogram showed reduced ejection fraction (EF) with structural changes and severe aortic stenosis. He was not a candidate for cardiothoracic surgery, and a transcatheter aortic valve replacement (TAVR) was performed. After the procedure, his symptoms improved, and during the outpatient follow-up, his cardiac function and dry weight improved.

Cardiac cachexia could be caused by reversible cardiomyopathy. Early assessment and approach are critical for the outcome of our patients, impacting their quality of life and outcome in terms of morbidity and mortality consequences.

## Introduction

Cachexia has been associated with multiple conditions: infectious, acute, and chronic conditions, including malignancies, cerebrovascular accidents, pulmonary disease, and heart failure (HF) [1,2]. Cachexia associated with heart failure is known as cardiac cachexia (CC). This definition has been an object of discussion [3]. There is no doubt that this complex condition represents a high mortality rate. Studies reported up to 40% one-year mortality in these patients in Europe [4,5]. This is a case of a patient who developed cachexia due to heart failure secondary to aortic stenosis (AS), which improved after valve replacement.

## Case presentation

The patient was a 68-year-old Hispanic male who presented to our office due to progressive shortness of breath limiting his daily activity (inability to walk more than 20 steps and to use stairs) and orthopnea, associated with a weight loss of 60 pounds in four months. He has a history of diabetes mellitus, severe aortic stenosis s/p valvuloplasty (seven years ago), and pulmonary edema due to heart failure decompensation. The physical examination showed a cachectic elderly male with an increased jugular venous pulsation; third and fourth sounds were also present. A telescopic aortic murmur was appreciated; his legs had pitting edema up to the hip, and his pulses were delayed.

**Figure 1:**
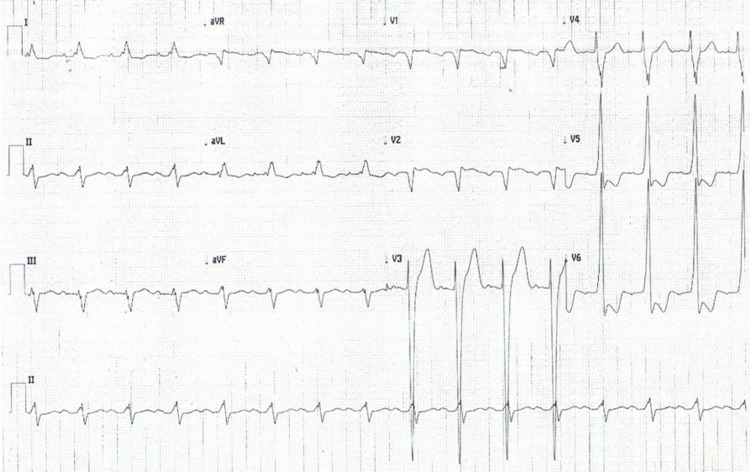
ECG at presentation showed normal sinus rhythm with LVH.

**Figure 2:**
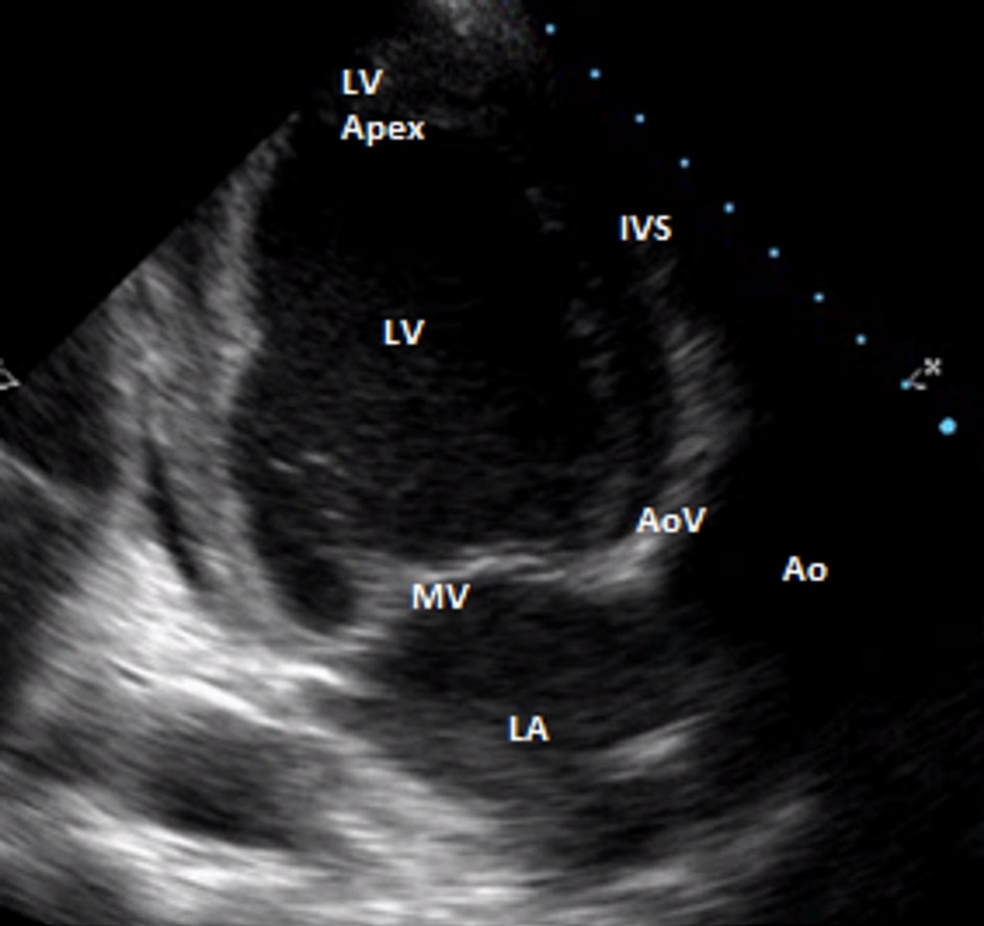
Echocardiogram showing parasternal long axis view. LV: left ventricle; IVS: interventricular septum; Ao: aorta; AoV: aortic valve; MV: mitral valve; LA: left atrium

His ECG showed left ventricular hypertrophy (LVH) with nonspecific ST changes (Figure 1), and the echocardiogram showed an ejection fraction (EF) of 20% with dyskinetic LV with dilation and hypertrophy (Figure 2). His aortic valve area was 0.8 cm^2^, peak gradient 51 mmHg, and mean gradient 30 mmHg. An angiotomography showed an aortic annulus of 25.95 mm (AVG) with an area of 5.25 cm^2^. Thyroid and autoimmune studies did not show any abnormalities, and he denied a history of dysphagia, odynophagia, epigastralgia, or rheumatic fever.

**Figure 3:**
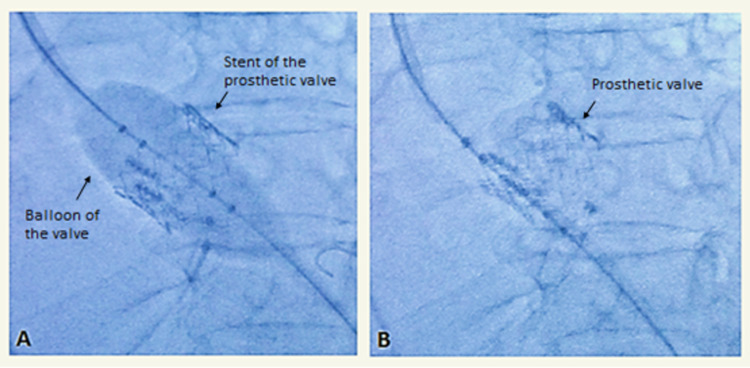
(A) Transcatheter prosthetic aortic valve deployment or implantation. (B) Post implantation.

This patient was not a candidate for cardiothoracic surgery, and a transcatheter aortic valve replacement (TAVR) was performed (Figure 3). He was discharged on aldactone, furosemide, aspirin, and clopidogrel.

After the procedure, the patient tolerated a supine position without orthopnea. During his follow-up visits, no pedal edema was noted, and his dry weight (10 pounds) increased after the procedure; also, his echocardiogram showed an ejection fraction of 46%.

## Discussion

In the elderly population, congestive heart failure (CHF) is the leading cause of morbidity and mortality, accounting for the majority of hospitalizations [6]. As the disease progresses, it can be associated with severe complications such as cardiac cachexia (CC), and this consequence is defined as at least 6% weight loss in six months [7]. A delay in the diagnosis might result in progressive weakness [8]. Deconditioning and fall injuries can lead to longer hospitalization, followed by death [9].

The prevalence of CHF is exponentially related to age, and it duplicates approximately every 10 years in males and every seven years in female patients [10]. This condition is a complicated interplay of many organ systems resulting in the hallmarks of cardiac cachexia, which are inflammation, wasting, atrophy of muscles, and multiorgan affections. Neuroendocrine and acute-phase reactant activation have been related to the progression of heart failure, as well as circulating proteins such as catecholamines, atrial natriuretic peptide, adiponectin, or heat-shock protein levels, which are also elevated in CC [11].

Cardiac cachexia is a pathological condition associated with worsening outcomes independent of other sociodemographic variables or CHF class [12]. It is described as a weight loss of 5% or more in the last 12 months (or a BMI < 20 kg/m^2^) in the presence of chronic illness in addition to three of the following: decreased muscle strength, fatigue, anorexia, low fat-free mass index, abnormal biochemistry characterized by increased inflammatory markers (C-reactive protein (CRP) and interleukin (IL)-6), hemoglobin < 12 g/dL, or hypoalbuminemia [13]. Anker et al. suggest that cardiac cachexia should be diagnosed when a weight loss of 6% or more is evident regardless of other criteria or other diseases [14].

Muscle wasting is an essential component of cachexia that usually precedes cachexia progression and predicts a worse prognosis in heart failure [15]. The exact mechanisms of cardiac cachexia induced by heart failure are not understood completely [16]. Possible etiologies include insufficient diet, malabsorption, metabolic dysfunction, urinary or gastrointestinal losses, and an abnormality between energy intake and expenditure, or its metabolism [17].

Another possibility in a patient with chronic CHF and tricuspid regurgitation is passive congestion of blood into the liver and intestines, which causes hepatomegaly and ascites, leading to decreased gastric capacity with feelings of abdominal fullness, nausea, satiety, and anorexia [18].

Patients with advanced heart failure have an activation of the catecholamines, pro-inflammatory cytokines, cortisol, and renin-angiotensin-aldosterone system (RAAS) that can further increase the metabolic rate and burning of more calories [19]. The effects of these pro-inflammatory cytokines promote proteolysis, cell death, and muscle and weight loss [20,21].

Angiotensin II and aldosterone are thought to be a factor in cellular changes, including death and fibrosis, through the activation of the ubiquitin-proteasome pathway [22]. Angiotensin increases tumor necrosis factor (TNF)-α, interleukin (IL)-6, serum amyloid A, glucocorticoids, and myostatin, which affect the synthesis and degradation of protein [23]. The catabolic state would then predispose these patients to develop cachexia due to the resting energy expenditure [24].

Improvement of bowel perfusion with agents such as angiotensin-converting enzyme (ACE) inhibitors (-)/angiotensin receptor blockers (ARBs) could assist in minimizing and preventing mucosal injury.

Other possible therapeutic options include agents that inhibit TNF-α such as pentoxifylline, reducing C-reactive protein through statins, and altering adiponectin or leptin levels [25].

Adequate nutrition in addition to diet, avoiding sedentarism, and regular exercise could avoid tissue wasting [8]. In educating patients with CHF and CC, it is essential to involve the relatives and community resources, including caregivers who need to appreciate the importance of nutrition and lifestyle modifications [26]. Psychosocial well-being is equally important [27].

Early diagnosis and prompt treatment of cardiac conditions are essential in the prevention of cardiac cachexia and its complications [26]. Valvular heart disease (VHD) is an essential cause of cardiovascular disease and complications in patients with aortic stenosis [28,29]. VHD is seen in 2.5% of the US population; it can be the primary cardiovascular risk for ventricular dysfunction due to the detrimental hemodynamic loading [30]. Severe aortic stenosis (AS) and HF represent a high risk of morbidity and mortality [31]. This group of patients with high risk for surgical intervention is referred for transcatheter aortic valve replacement (TAVR) [32].

The most crucial point is distinguishing between primary heart failure and AS versus HF due to other etiologies, including severe ischemic cardiomyopathy [33,34]. Suppose HF is predominantly caused by excessive afterload as in AS [35]. In that case, the chance of left ventricular (LV) improvement after TAVR is high if it is done promptly [36]. However, LV improvement after the intervention is uncertain in the presence of scarring due to ischemia [36].

In AS, the LV will respond to the elevation of the pressure load with adaptive concentric wall hypertrophy keeping wall stress and LVEF [37]. In advanced AS, the pressure cannot be managed by LV hypertrophy, and it is associated with poor outcomes [38].

Aortic outflow obstruction can be relieved mechanically via surgical aortic valve replacement (SAVR), which can improve symptoms, LV function, and outcomes in patients with advanced AS. However, the prognosis of SAVR depends on ventricular function prior to the surgery. In patients with ventricular function impairment or poor surgical candidates, SAVR is associated with high perioperative mortality and morbidity [39].

Reduced LVEF has been associated with increased operative mortality risk. According to the Euro Heart Survey, up to one-third of the patients with severe AS and reduced LVEF was deemed not acceptable for surgery. However, TAVR has changed the management of nonsurgical candidates with AS. TAVR has evolved to the standard in high-risk patients that are not suitable for surgery. Moreover, TAVR is related to more significant ventricular function improvement compared with surgical patients due to the lack of stress during the procedure that helps avoid inflammation, ischemia, and oxidative injury, which lead to apoptosis and dysfunction [40,41].

Literature has established the improvement of EF and outcomes after valve replacement due to aortic stenosis [42,43].

## Conclusions

Cardiac cachexia could be caused by reversible cardiomyopathy. Besides, it is poorly understood; it plays an essential role in the outcome of our patients and leads to potential complications in heart failure patients. Early assessment and approach are critical for our patients, impacting their quality of life, and morbidity and mortality, the reason why these patients should be closely followed and their nutritional status needs to be monitored frequently. 
